# MgZnO/ZnO heterostructures with electron mobility exceeding 1 × 10^6^ cm^2^/Vs

**DOI:** 10.1038/srep26598

**Published:** 2016-05-27

**Authors:** Joseph Falson, Yusuke Kozuka, Masaki Uchida, Jurgen H. Smet, Taka-hisa Arima, Atsushi Tsukazaki, Masashi Kawasaki

**Affiliations:** 1 Department of Applied Physics and Quantum-Phase Electronics Center (QPEC), The University of Tokyo, Tokyo 113-8656, Japan; 2 Department of Advanced Materials Science, The University of Tokyo, Kashiwa 277-8561, Japan; 3 Max Planck Institute for Solid State Research, 70569 Stuttgart, Germany; 4 RIKEN Center for Emergent Matter Science (CEMS), Wako 351-0198, Japan; 5 Institute for Materials Research, Tohoku University, Sendai 980-8577, Japan; 6 PRESTO, Japan Science and Technology Agency (JST), Tokyo 102-0075, Japan

## Abstract

The inherently complex chemical and crystallographic nature of oxide materials has suppressed the purities achievable in laboratory environments, obscuring the rich physical degrees of freedom these systems host. In this manuscript we provide a systematic approach to defect identification and management in oxide molecular beam epitaxy grown MgZnO/ZnO heterostructures which host two-dimensional electron systems. We achieve samples displaying electron mobilities in excess of 1 × 10^6^ cm^2^/Vs. This data set for the MgZnO/ZnO system firmly establishes that the crystalline quality has become comparable to traditional semiconductor materials.

Reducing the dimensionality of an electrically conducting system is known to have a dramatic effect on the nature of the observable physical phenomena. In the case of two-dimensional systems, where charge carriers are free to move in the *x* − *y* directions but confined spatially in the *z*, a vast array of ground states^1^ and non-equilibrium^2^ effects have been revealed, concomitant to improvements in material quality. A figure often used to gauge such quality is the carrier mobility (*μ*). In the Drude picture of DC conductivity, this value probes large momentum loss scattering events and is reflected in the sample’s longitudinal resistivity *ρ*_*xx*_ = 1/*μen*, where *e* is the elementary charge and *n* is the charge carrier density. At low temperature, once the effects of phonon scattering have been mitigated, this value may reach exceptionally high values. In the case of the AlGaAs/GaAs two-dimensional electron systems (2DES), mobilities beyond 3 × 10^7^ cm^2^/Vs have revealed a plethora of exotic phenomena at low temperatures^3^,^4^,^5^. Responsible for these advances is the molecular beam epitaxy (MBE) apparatus; the work-horse of ultra-high purity thin film growth, whereby the evaporation of high purity source materials allows the control of film composition and structure on an atomic scale as designed. The nature of the disorder limiting *μ* in state-of-the-art AlGaAs/GaAs 2DES is the back-ground impurity content of films^6^, despite samples being grown using source materials with purity of roughly 7N and using apparatus manufactured from refractory metals. The adaptation of MBE to the field of oxide thin film growth has similarly resulted in higher quality crystallinity compared with other growth techniques, in turn enabling the exploration of the rich degrees of freedom available^7^,^8^,^9^,^10^.

The deployment of this technique to the growth of the relatively simple chemical and crystallography natured ZnO is the focus of this report. We present the current state-of-the-art growth apparatus and techniques for achieving high quality Mg_*x*_Zn_1−*x*_O/ZnO heterostructures which host a 2DES at the heterointerface^11^,^12^. Similar to AlGaN/GaN heterostructures^13^, the polar nature of the inversion asymmetric Wurtzite structure of ZnO is exploited for the formation of a 2DES, without the need for remote doping techniques. As a result, long range potential fluctuations typically imposed by such doping techniques are absent, with short range scattering events relatively dominant^14^. This scenario is different to the AlGaAs/GaAs 2DES and therefore these ZnO 2DES allow for the exploration of alternate facets of the interplay between the types of disorder and observable physical phenomena. Here we target individual scattering origins through a systematic study of growth conditions and sample design.

## Results

Previous reports^10^,^12^,^15^ on this system have revealed that the 2DES charge density depends intimately on the Mg content of the capping Mg_*x*_Zn_1−*x*_O layer down to dilute concentrations *x* ≤ 0.01. In the following sections, we address prominent impurity sources within the oxide MBE apparatus and refine the growth conditions, which, in combination with tuning the Mg content, enable reproducible growth of films with *μ* exceeding 1 × 10^6^ cm^2^/Vs. While this *μ* occurs for only a narrow charge density range, significant enhancements in *μ* are achieved for the full range of *n*. We also gain access to dilute samples where *n* = 2.1 × 10^10^ cm^−2^.

### Contamination sources

**Figure 1:**
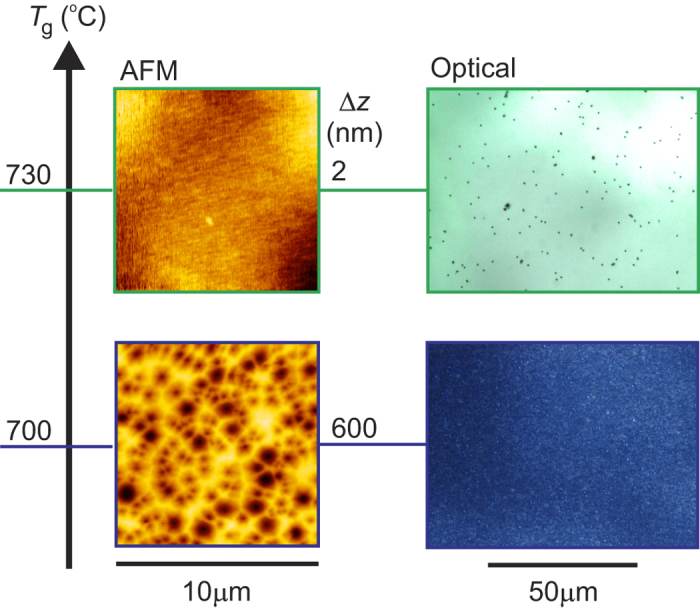


We begin by addressing some technical aspects of the MBE apparatus which have evolved. Their implementation has been a prerequisite for enabling the results of this work. The basic configuration of the apparatus is presented in the methods section. Many technical aspects are common with ref. 15, including the use of pure ozone as the oxygen source, while the purity of Zn has been improved from 7N to beyond 7N5, where quantitative analysis of impurities becomes commercially unviable. In addition to the inherently lower impurity content of ozone compared to oxygen radical cells^15^, ozone as an oxygen source offers the benefit of an expanded temperature window for uniform growth. This is important as the presence of oxygen simultaneous to high temperatures leads to stringent requirements in apparatus design and handling to avoid increased impurities being expelled. Previous works using oxygen radical cells^16^ found the minimum growth temperature to achieve a smooth sample surface to be roughly *T*_g_ ≥ 810 °C under similar Zn flux conditions. As illustrated in Fig. 1, the temperature range is expanded through the use of ozone down to *T*_g_ ≥ 730 °C. In this figure, the left column of panels displays the surface morphology measured by atomic force microscopy (AFM) while the right column depicts an optical microscope image. Notably, in the *T*_g_ = 700 °C film, the surface is covered by an overwhelmingly high density of pits. The higher temperature films display clean local surface morphology, as measured by AFM, and a relatively low density of macroscopic defects (mostly pits) in optical microscopy. We have not been successful in completely eliminating the finite density of pits despite performing wet-etching and annealing of each substrate prior to the growth^17^. These originate from the substrate surface^15^ and are likely due to point defects generated during the manufacturing process which prohibit epitaxial growth.

The presence of oxygen within the chamber places limitations on the suitability of refractory metals (W, Mo, Ta). These are widely used in traditional MBE chambers, but their oxides may be highly volatile and therefore contaminate grown films. In light of this, we have extensively employed superalloys for hot components. Specifically, we have found the nickel-based super-alloy Haynes®-230, and to a lesser extent Inconel®-601 to be useful in the heating apparatus itself and substrate holders. We also have endeavoured to eliminate pyrolytic boron nitride (pBN) components from the main substrate heating unit, as a peak of 28 amu (corresponding to what is thought to be N_2_) was resolved in the residual gas analysis of the chamber when the indicated temperature of the heating coil exceeded 1000 °C. This was accompanied with a strong suppression of *μ*. The ability to go to lower growth temperature with ozone has therefore mitigated the impurities originating from the heating unit. As an additional precaution, the pre-growth substrate anneal temperature too has been lowered from ≈1000 °C to *T*_g_ itself. Currently, the heating apparatus consists of a pure SiC coil with a diameter of 80 mm encased in a super-alloy construction. Only electrical insulating components have been made out of pBN. The coil itself remains oversized relative to the size of the substrates in use and therefore further reductions in impurity content may be achieved through downsizing the coil^18^, or replacement by a long wavelength laser heating system^19^ which would eliminate the need for pBN electrical isolators altogether.

Equally crucial has been the choice of substrate holder material, as this part is exposed to particularly strenuous conditions due to the direct and continual ozone flow and high temperatures. Previous studies using Inconel® substrate holders found Mn impurities originating from the holder itself within grown films in secondary ion mass spectroscopy (SIMS)^20^. These were eliminated by using quartz holders in combination with high *T*_g_ (≈920 °C). However, as discussed above, this heralds the inclusion of impurities from the heating apparatus. To overcome this, we again employ superalloys for the substrate holder and find that the content of atomic species that are unintentionally incorporated in grown films as impurities is below the detection limit of SIMS. The use of superalloy substrate holders also results in roughly half the power dissipation (≈250 W) in comparison with the use of quartz. We have however identified two scenarios which lead to disastrous results and significant impurities. These encompass both the choice and handling of the substrate holder forming superalloy. We have found that hundreds of samples maybe grown consecutively using substrate holders made from Haynes®-230, with grown films remaining clean to the edge of the substrate. Impurities from the alloy itself can not be detected in SIMS. In contrast, films grown with Haynes®-214 either display a high density of pit-defects on the sample edges, or are completely insulating at cryogenic temperatures. This occurs in spite of the superior oxidation resistance of the Al-rich protective layer (referred to as the oxide scale) of Haynes®-214 at high temperature according to manufacturer specifications.

**Figure 2:**
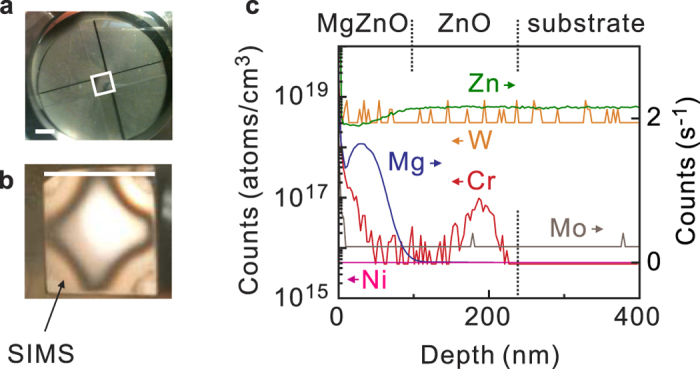
(**a**) Photograph of the underside of an oxidized Haynes®-230 substrate holder. The opening is indicated by a white square and hosts the 1 × 1 cm ZnO wafer. (**b**) a post-growth photograph of a wafer which has been grown following descaling of the substrate holder. The white scale bar is 1 cm for both photographs. The arrow points to the section of the film which is analysed by SIMS. The result is shown in (panel **c**), where Cr impurities are detected in the grown MgZnO and ZnO layers.

Even when selecting a suitable material such as Haynes®-230, it is crucial to maintain the original protective oxide scale formed from the virgin alloy after initial use. A photograph of the underside of the substrate holder is displayed in Fig. 2a and shows a typical scale after multiple growths. The hole in the middle demarcated by a white box is where the substrate is placed. The green tinge is the scale and is primarily composed of Ni and Cr oxides. This layer may be removed through exposure to nitric acid, returning it to a metallic appearance (not shown). However, films grown following such a procedure are known to display surface morphologies like that shown in panel b, where the corners are of a clouded visual appearance due to a high density of pits. Measuring SIMS locally in this region yields the depth profile in panel c. Up to the substrate the impurity density is below the detection limit. However entering the homoepitaxial ZnO layer, a large (10^17^ atoms/cm^−3^) Cr signal is resolved. While this content is far below the solubility limit of Cr in ZnO^21^, it evidently has a negative impact on the ability to form an abrupt step-like distribution of Mg, which is representative of the MgZnO barrier and hence the 2DES-hosting quantum well. It may be that a different phase, perhaps spinel ZnCr_2_O_4_ or Cr_2_O_3_, forms microscopically here and inhibits the proceeding growth. Accordingly, such regions are insulating at cryogenic temperatures. It is therefore only if the original oxide scale is retained that a substrate holder may be used hundreds of times to deliver consistently high mobility samples, whereas the removal of the scale has irreversible and detrimental consequences.

**Figure 3:**
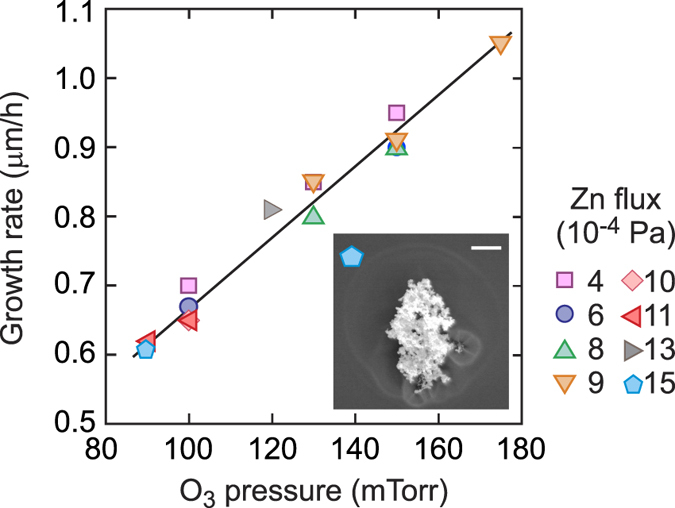
Growth rate as a function of O_3_ pressure measured by the baratron neighbouring the piezo leak valve. The linear interpolation is a guide for the eye. The inset displays scanning electron micrograph of a surface defect. Scale bar = 1 μm.

We now set the growth temperature to be *T*_g_ = 750 ± 10 °C and explore the growth conditions. In line with previous reports^15^, all films are grown under Zn rich conditions. This is conveyed in Fig. 3 where the growth rate of films is shown to depend solely on the O_3_ flow across a wide range of Zn flux pressures. The ozone flow is read by a capacitive baratron gauge near the leak value and the Zn flux by a naked ion gauge placed at the location of the substrate (see methods for details). The samples are comprised of both a ZnO homoepitaxial and Mg_*x*_Zn_1−*x*_O capping layer (*x* ≈ 0.01). At such *x* content, it is not possible to detect a difference in growth rate for the ZnO and Mg_*x*_Zn_1−*x*_O layers. For all growth rates the pit density is comparable, however when reducing the ozone flow and increasing Zn flux a number of macroscopic defects emerge on the surface, as shown in the inset for a representative sample grown with a Zn flux of 15 × 10^−4^ Pa.

### Film thickness

**Figure 4:**
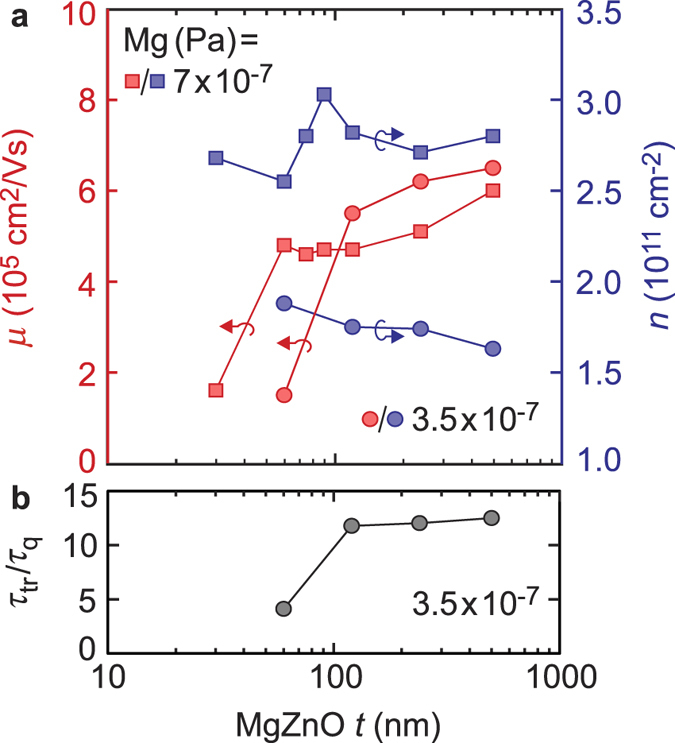
(**a**) *μ* and *n* as a function of MgZnO capping layer thickness for two sets of samples corresponding to Mg flux of 3.5 (circles) and 7 × 10^−7^ Pa (squares). (**b**) The ratio of transport (*τ*_tr_) and quantum (*τ*_q_) scattering times for samples grown under 3.5 × 10^−7^ Pa flux conditions.

We now return to *μ* as a gauge of the quality of the 2DES at the heterointerface. Figure 4 explores the effect of the thickness (*t*) of the MgZnO layer. For all films the growth conditions are kept constant (Zn ≈ 10 × 10^−4^ Pa, O_3_ = 90 mTorr). A 500 nm homoepitaxial layer of ZnO is deposited first and is capped with a MgZnO layer of *t* varied between 30 ~ 500 nm by controlling the growth time. Two data sets are presented in panel a; Mg flux = 3.5 and 7 × 10^−7^ Pa. Even for the thinnest film of *t* = 30 nm charges accumulate to form the 2DES, in agreement with previous reports for higher density samples^12^. However, it can be seen that *μ* is greatly suppressed as *t* is made thinner for both datasets. Moreover, this suppression occurs at a thicker *t* for the lower density samples. In panel b the ratio of transport (*τ*_tr_) and quantum (*τ*_q_) scattering times are presented for this dilute dataset (here *T* = 40 mK). This ratio can aid in differentiating between short- or long-range scattering limited mobilities, with a lower ratio indicating short-range scattering is dominant. A similar, more extensive analysis has been published in ref. 14 and we refer the reader to this for details of the analysis. This ratio reduces with lower *t*, suggesting a trend towards enhanced short-range scattering. We recall that no intentional modulation doping is present in the MgZnO/ZnO heterostructure, as opposed to the AlGaAs/GaAs system, which may screen surface disorder. The data therefore conveys that the surface of the heterostructure is a prominent source of disorder, and, while an increased density of the 2DES acts to screen this, bringing this close to the 2DES drastically affects not only the magnitude but also nature of scattering.

### Flux ratios

**Figure 5:**
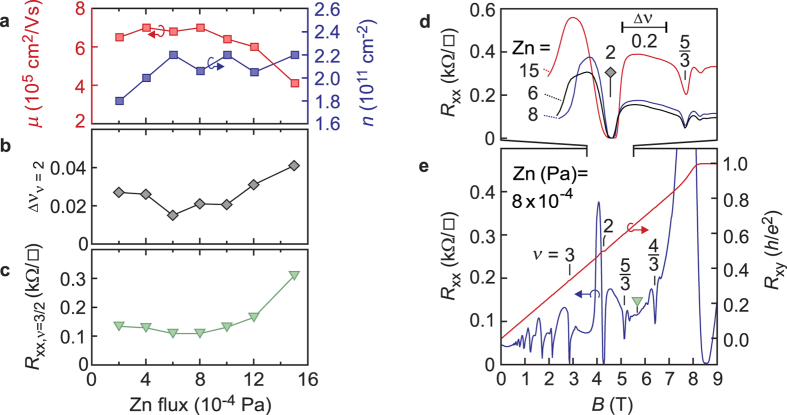
Zn flux dependence of (**a**) *μ* and *n*, (**b**) the width of the *ν* = 2 integer quantum Hall state in *R*_*xx*_, (**c**) *R*_*xx*_ at *ν* = 3/2 and (**d**) the magnetotransport around *ν* = 2. (Panel **e**) displays the magnetotransport for a film grown with Zn flux = 8 × 10^−4^ Pa. All data corresponds to *T* = 500 mK, with O_3_ pressure = 90 mTorr and thickness = 500 nm of MgZnO.

We now set *t* = 500 nm, Mg flux = 5.5 × 10^−7^ Pa and 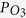 = 90 mTorr in order to explore the characteristics of heterostructures as a function of Zn flux (*i.e.* Zn cell temperature). As conveyed in Fig. 3, all film growth takes place under Zn rich conditions. However, ZnO is notorious for defect formation whose exact nature remains under debate^22^,^23^,^24^. It results in finite room temperature conductivity, despite the large band-gap of the bulk. Figure 5a–c confirms that the exact ratio of Zn and O_3_ affects the 2DES. While a rather forgiving window is observed, both deficient (Zn ≤ 4 × 10^−4^ Pa) or over-rich (Zn ≥ 10 × 10^−4^ Pa) Zn flux conditions suppress *μ* (panel 5a), while *n* is not significantly affected. When the flux is increased beyond 10 × 10^−4^ Pa a significant density of particles develops on the surface, as shown in the inset in Fig. 3 for the case of flux = 15 × 10^−4^ Pa. These occur in addition to a finite density of pits ubiquitous in each growth. The suppression of *μ* in the high Zn flux regime is likely a consequence of both these particles as well as an increase in defects encountered within the crystal structure.

Quantifying the quality of 2DES samples beyond *μ* remains a controversial and formidable process. This is evidenced in the case of the *ν* = 5/2 fractional quantum Hall (FQH) state which, in AlGaAs/GaAs heterostructures^4^,^25^,^26^, shows only a loose correlation with *μ*. While a full exploration between sample parameters and the stability of correlated ground states is beyond the scope of this work, we lay the foundations by exploring the nature of transport observed at the standard characterization temperature of *T* = 500 mK. While 5a solely focused on *μ* for evaluating samples, we present two more facets of analysis; the width of the *ν* = 2 integer quantum Hall state minimum in *R*_*xx*_ in 5b and *R*_*xx*_ at *ν* = 3/2 in 5c. The motivation to address the first quantity stems from a correlation that has been established between the amplitude of the density variations in the sample and plateau widths in experiments that have addressed the microscopic origin of localization in the quantum Hall regime by measuring the local compressibility^1^,^27^. Narrower integer quantum Hall minima should therefore reflect a cleaner 2DES. The second quantity, the resistance at an even denominator fractional filling, measures the mobility of composite fermions, which are responsible for the formation of correlated ground states at fractional fillings^1^. In comparison with the electron mobility derived from the zero field resistivity this quantity should reflect more directly their backscattering probability. The lower the resistivity at this fillings the more pronounced fractional quantum Hall states are expected to be. It has been used to predict the quality of the *ν* = 5/2 ground state in AlGaAs/GaAs heterostructures^5^. In MgZnO/ZnO heterostructures filling 3/2 has the unique potential of hosting an even-denominator fractional quantum Hall state through sample rotation^28^.

A typical transport trace of a heterostructure is shown in 5e, for a film of Zn flux = 8 × 10^−4^ Pa. At this temperature, both the integer and FQH effect are observed, allowing a study of their correlation with *μ*. A close-up of the *ν* = 2 state is shown in 5d for a number of films growth with different Zn fluxes. We avoid plotting the data on a *B*-field axis as *n* varies slightly (~10%) between films, instead plotting it on *ν*. The width of the zero resistance minimum is referred to as Δ*ν*. Its Zn flux dependence is plotted in panel b for the same set of films as in panel a. A good correlation is observed with *μ* of the films; the highest *μ* samples show the sharpest integer states (lowest Δ*ν*). As shown in panel c, the lowest resistance at *ν* = 3/2 is achieved concomitant to the peak *μ* and narrowest *ν* = 2 state. The above analysis suggests a significant correlation between *μ* and other facets of the magnetotransport available at *T* = 500 mK used to gauge quality. This is distinct from the occasional lack of correlation between mobility and quality that has been reported on AlGaAs/GaAs heterostructures^4^,^29^.

**Figure 6:**
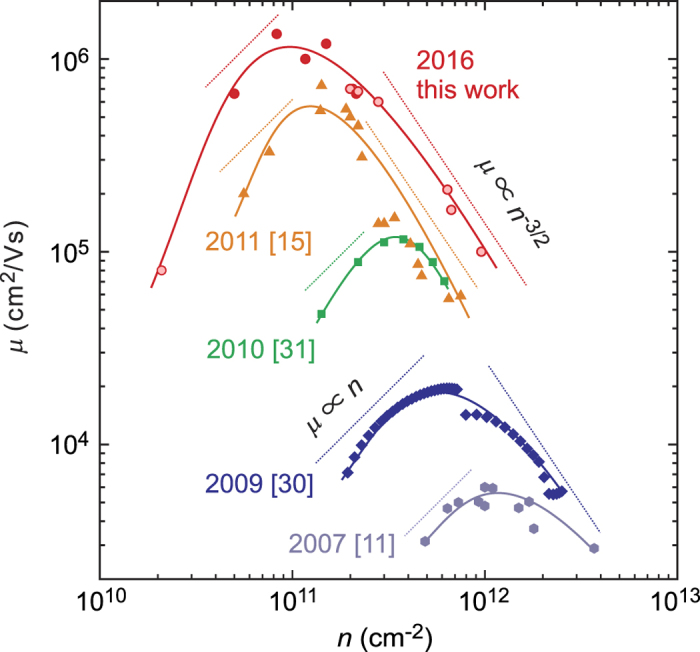
Mobility as a function of density for historical growth regimes (For all data *T* ≤ 2 K). Data is adapted from refs 11, 15, 30 and 31. For this work, bold symbols correspond to *T* ≤ 100 mK while lightly shaded are for *T* = 500 mK. The guidelines given by *μ* ∝ *n* and *μ* ∝ *n*^−3/2^ for each data set represent charged impurity scattering and interface/alloy scattering limitations to the mobility.

The analysis also allows us to determine that the most suitable conditions occur at Zn flux = 6 ~ 8 × 10^−4^ Pa when the ozone flow is set to 90 mTorr. Having established these growth parameters we vary *x* in Mg_*x*_Zn_1−*x*_O to modify the charge density^15^. The dataset is presented in Fig. 6 where *μ* is plotted as a function of *n*. Data adapted from refs 11, 30 and 31 are included to illustrate the historical advances achieved. This data must be interpreted taking in mind that the temperature of each data set varies, but all are for *T* ≤ 2 K and generally represent the saturated mobility. The values presented for the data set of this work have been accumulated at *T* ≤ 500 mK, since *μ* saturates at increasingly lower temperatures for samples of *n* ≤ 1.5 × 10^11^ cm^−2^ (ref. 15). In fact, *T* ≈ 100 mK are required for dilute samples, necessitating the use of a dilution refrigerator. The highest *μ* film is achieved for *n* = 8.3 × 10^10^ cm^−2^ and displays 1,300,000 cm^2^/Vs. A mobility in excess of 1,000,000 cm^2^/Vs may be maintained even when increasing *n* to 1.5 × 10^11^ cm^−2^. For both lower and higher *n* from this range, *μ* is suppressed due to different scattering mechanisms; insufficient screening of impurities within the crystal structure in the case of dilute films, and increased interface roughness or alloy scattering induced by the random potential of Mg content in higher density films. The analysis performed in ref. 14 corroborates such scattering mechanisms as being prominent for each regime. In Fig. 6 we also include reported scaling behaviour as guidelines corresponding to charged impurity scattering (*μ* ∝ *n*) and interface/alloy scattering limitations (*μ* ∝ *n*^−3/2^) for each data set^32^. Ultimately, according to Matthiessen’s rule, the interplay between these two mechanisms determines the maximum mobility which may be achieved. In comparison to historic data, the maximum *μ* of films has occurred at decreasing densities. This is a reflection of the reduced impurity content achieved, with the employment of O_3_ as an example. Less dramatic gains have been achieved in high density samples, reflecting the difficulty inevitably encountered in mitigating the detrimental effect the random distribution of Mg atoms in the MgZnO alloy has. Mg is of course inherently required to induce the 2DES in the first place. Finally, we note that conducting samples of density *n* = 2.1 × 10^10^ cm^−2^ have been achieved. This *μ* is for *T* = 500 mK, as below this a surprising upturn in resistance occurs, whose nature remains to be understood, but may indicate the transition to a new type of electronic ground state at low temperature.

## Discussion and Conclusions

**Figure 7:**
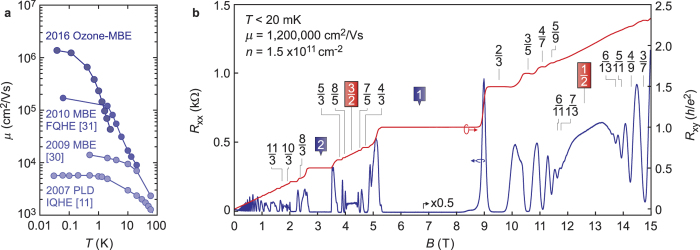
(**a**) Temperature dependence of *μ* for recent generations of MgZnO/ZnO heterostructures. Data is representative samples of refs 11, 30 and 31. (**b**) Low temperature (*T* < 20 mK) magnetotransport of a high mobility sample.

In this work we have described in detail technical aspects and growth conditions utilised in the ozone-assisted MBE growth of high mobility MgZnO/ZnO heterostructures. Each data set presented aims to identify unique sources of disorder. Addressing these culminates in the realization of heterostructures which display *μ* in excess of 10^6^ cm^2^/Vs. We conclude this work by summarising the gains in Fig. 7a where the peak *μ* for respective historical growth regimes and the physics they enabled, namely the integer quantum Hall effect (IQHE) and fractional quantum Hall effect (FQHE), is displayed as a function of temperature using a similar representation to refs 3 and 33. Steady technological advances have enabled each step above the previous. Namely, the introduction of MBE as a replacement to pulsed laser deposition (PLD) between refs 11 and 30. Further refining the MBE growth conditions while still retaining oxygen plasma as the oxidizing species enabled the data in ref. 31. The use of ozone in ref. 15 reduced the residual impurity content of films and hence boosted the mobility significantly by enabling lower density ad therefore lower Mg content samples. Finally, refining the growth conditions using ozone in addition to the other technical advances detailed in this work gives the current data set. As analysed in ref. 14 in detail, the current generation of samples display *τ*_tr_ (≈600 ps) and *τ*_q_ (≈30 ps) comparable to AlGaAs/GaAs heterostructures of similar charge density. The magnetotransport of a heterostructure of *μ* = 1,200,000 cm^2^/Vs and *n* = 1.5 × 10^11^ cm^−2^ is shown in 7b at *T* < 20 mK. A rich array of FQH states is revealed at this temperature, including those in the lowest Landau level (*ν* < 2), where FQH sequences centred on both *ν* = 1/2 and 3/2 are observed. They are consistent with the hierarchy predicted by the composite fermion model^1^. In addition, states at higher filling factors (*ν* > 2) are resolved and are consistent with the conclusions drawn in ref. 28. We finally note that the Wigner-Seitz radius (*r*_*s*_) of the dilute (*n* ≈ 2 × 10^10^ cm^−2^) yet high mobility (*μ* ≈ 100,000 cm^2^/Vs) films realised in this work approaches values of 30 (assuming an effective mass of m^*^ = 0.3 *m*_0_). In such a parameter space the ground state of a 2DES is predicted to show instability towards ferromagnetism or even Wigner crystallization^34^, which may account for the observed increase in resistance for *T* ≤ 500 mK. Exploring this entails the need for detailed *I* − *V* characteristics, as well as an understanding of the spin susceptibility of the 2DES, perhaps through rotation and/or electron spin resonance techniques. This opens the exciting avenue to study highly correlated electron physics in an *r*_*s*_ regime previously inaccessible with heterostructures of this quality level.

## Methods

Samples have been grown using an Epiquest RC2100 MBE chamber of base pressure 1 × 10^−8^ Pa when the liquid nitrogen jacket is filled. Single crystal Zn-polar [0001] ZnO substrates supplied by Tokyo Denpa of resistivity *ρ* ≤ 4 Ωcm were utilised. Low resistivity substrates were exclusively used due to their lower residual Li content resulting from their growth via hydrothermal method. These are prepared by dipping in HCl:H_2_O = 7:200 solution as described in ref. 17 and then heated to 200 °C within the MBE load-lock chamber (of pressure ~1 × 10^−5^ Pa). This temperature was increased from 150 °C used in previous studies after replacing the heating apparatus with a more efficient tungsten coil. Once inside the growth chamber, the substrate is gradually heated to *T*_g_ over a 30 minute time span and then held for 30 minutes under high vacuum before flux is introduced. The power dissipation of the heating unit is roughly 250 W. Two Zn (7N5) and two Mg (6N) conventional effusion cells are equipped and provide metallic flux from opposing directions within the growth chamber. The typical working temperatures for all cells is about 270 °C with a power dissipation of roughly 40 W. For the Zn flux, both cells are adjusted to be equal and used simultaneously whereas for Mg only one cell is opened. A Zn flux of 1.6 × 10^−4^ Pa resulted in a measured deposition rate of 1 Å/s of metal, corresponding to a flux of 6.5 × 10^14^ atoms/cm^2^ s. Liquid ozone is generated by a Meidensha MPOG-104A1-TH pure ozone generator and fed into the MBE chamber via a PID controlled piezoelectric leak valve (Oxford Applied Research PLV1000) where the pressure is read by a capacitive baratron and a feedback loop is established. O_3_ is delivered to the vicinity of the substrate through a Veeco low temperature gas cell set at *T* = 80 °C encased within a water cooled jacket. The exact purity of O_3_ reaching the substrate is unknown but should be close to 100% while within the gas lines. The substrate is rotated at a speed of 7 rpm during growth to enhance the uniformity of the sample^35^. The substrate temperature is monitored by both a thermocouple located at the centre of the heating coil and an infrared camera (detecting 8 μm wavelength radiation) located outside the chamber, which monitors the local substrate temperature, which we define as *T*_g_, through a BaF_2_ viewport. The thickness of films is determined by a surface profiler, as the dilute Mg content in most films prevents an analysis via X-ray diffraction^36^. The exact Mg content for each film has not been investigated for similar reasons, but may be estimated through the relationship *n* = 1.5 *x* × 10^13^ cm^−2^ (ref. 10). Electrical measurements were performed using the van der Pauw method, where raw samples were cut into roughly 3 × 3 mm chips and then indium electrodes soldered at the corners. The high uniformity of Mg as a result of rotation during the growth allows the study of such large samples^35^. These were performed at temperatures *T* ≤ 500 mK in ^3^He and dilution refrigerators.

## Additional Information

**How to cite this article**: Falson, J. *et al.* MgZnO/ZnO heterostructures with electron mobility exceeding 1 × 10^6^ cm^2^/Vs. *Sci. Rep.* **6**, 26598; doi: 10.1038/srep26598 (2016).
